# A Case of False-Positive* Mycobacterium tuberculosis* Caused by* Mycobacterium celatum*

**DOI:** 10.1155/2016/1761923

**Published:** 2016-11-08

**Authors:** Edward Gildeh, Zaid Abdel-Rahman, Ruchira Sengupta, Laura Johnson

**Affiliations:** ^1^Department of Medicine, Henry Ford Hospital, Detroit, MI 48202, USA; ^2^Department of Infectious Diseases, Henry Ford Hospital, Detroit, MI 48202, USA

## Abstract

*Mycobacterium celatum* is a nontuberculous mycobacterium shown to cause symptoms similar to pulmonary* M. tuberculosis*. Certain strains have been shown to cross-react with the probes used to detect* M. tuberculosis*, making this a diagnostic challenge. We present a 56-year-old gentleman who developed signs and symptoms of lung infection with computed tomography scan of the chest showing right lung apex cavitation. Serial sputum samples were positive for acid-fast bacilli and nucleic acid amplification testing identified* M. tuberculosis* ribosomal RNA, resulting in treatment initiation. Further testing with high performance liquid chromatography showed a pattern consistent with* M. celatum*. This case illustrates the potential for* M. celatum* to mimic* M. tuberculosis* in both its clinical history and laboratory testing due to the identical oligonucleotide sequence contained in both. An increasing number of case reports suggest that early reliable differentiation could reduce unnecessary treatment and public health intervention associated with misdiagnosed tuberculosis.

## 1. Introduction

*Mycobacterium celatum* was first described in humans in 1993 [1]. It is a member of the slow-growing nonchromogens, nontuberculous mycobacterium, which exists along with 150 other currently identified rapid-growing and slow-growing species [2]. It has been shown to cause fatal disease in both immunocompetent and immunocompromised patients.

*M. celatum* and* M. tuberculosis* can present with a similar clinical picture: cough, weight loss, lung infiltrates, or cavitary lesions; thus, differentiation between these 2 organisms can be difficult. Although culture remains the gold standard for diagnosis, identification using nucleic acid amplification testing (NAAT) provides a much faster answer and saves a considerable amount of time. It is highly sensitive [3] and allows for faster initiation of therapy weeks before definitive culture results return. A challenge exists with diagnosing this type of mycobacterium, as it has been found to cross-react with the current probes used to diagnose* M. tuberculosis*.

Here we describe a case of a middle-aged immunocompromised patient who was misdiagnosed and treated for tuberculosis but ultimately was found to have* M. celatum*.

## 2. Case Presentation

Our patient is a 56-year-old man with medical history significant for common variable immune deficiency on biweekly intravenous immunoglobulin therapy who presented initially to Henry Ford Hospital for evaluation for fecal transplantation for* Clostridium difficile* diarrhea refractory to medical management. The patient underwent his first fecal transplant with transient improvement, but his diarrhea recurred and he subsequently failed 3 further fecal transplants. He was finally initiated on long-term oral vancomycin with symptom resolution.

**Figure 1:**
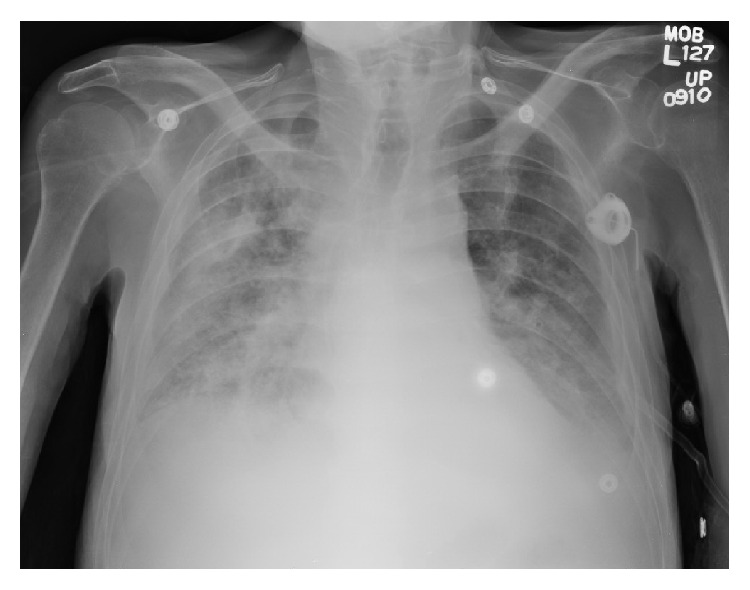
Chest X-ray showing diffuse consolidation throughout the right lung.

His hospital course was complicated by increasing oxygen requirements from a baseline of 2 L/min at home to 6 L/min. Lung auscultation revealed diffuse rhonchi and decreased breath sounds on the right side. He was afebrile and had increasing low to normal white blood cell counts in the prior 3 days (2.6–6.0 × 10^9^/L). Chest X-ray showed diffuse consolidation throughout the right lung consistent with pneumonia (Figure 1), and he was started on intravenous antibiotics for treatment of hospital-acquired pneumonia.

**Figure 2:**
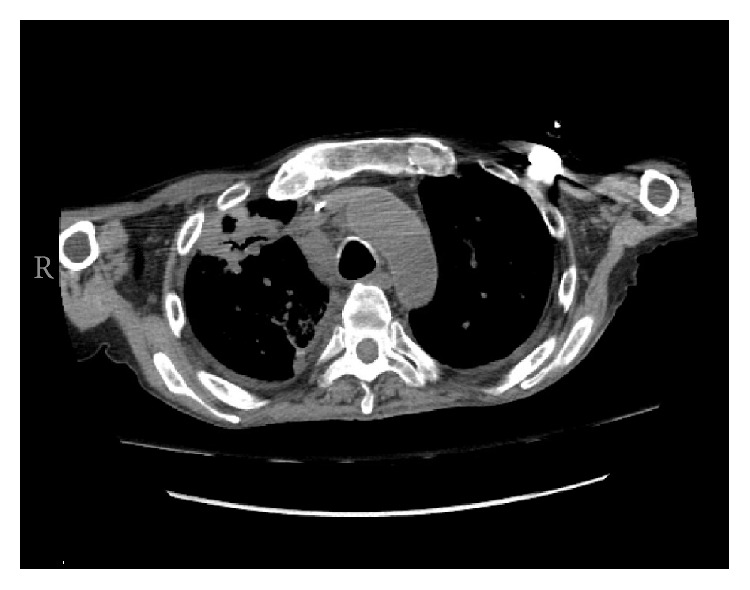
Chest computed tomography scan showing cavitary lesion in the right lung apex.

Three days after initiating therapy, the patient still remained short of breath and hypoxic. Computed tomography scan of the chest was obtained, which showed consolidation within the right lung apex with areas of central lucency suggesting cavitation (Figure 2).

Serial sputum samples were positive for acid-fast bacilli, and NAAT (Enhanced Amplified* Mycobacterium tuberculosis* Direct Test, Gen-Probe, San Diego, CA) run by the state of Michigan, identified* M. tuberculosis* complex ribosomal RNA, providing the diagnosis of tuberculosis. The patient was started on isoniazid, ethambutol, pyrazinamide, and rifampin while awaiting results of cultures.

A second non-NAAT test (AccuProbe, Hologic, Marlborough, MA) was run and came back negative for tuberculosis. Two weeks later, high performance liquid chromatography showed a pattern consistent with* M. celatum*, and the 4-drug regimen was changed to ethambutol, azithromycin, and moxifloxacin. Final cultures confirmed the result.

## 3. Discussion

*M. celatum* is a slow-growing, nontuberculous mycobacterium that is morphologically similar to* Mycobacterium avium* complex [3]. It can be divided into 3 types, 1, 2, and 3, based on their genomic sequencing. Previous reports have shown cross-reactivity between* M. celatum* types 1 and 3 and the genetic probe for the* M. tuberculosis* complex. The purpose of this correspondence is to add to the literature supporting this phenomenon and to report it in a CVID patient and summarize the current evidence.

*M. celatum* has been classically described in patients with acquired immunodeficiency syndrome [4], with less than a handful of cases described in immunocompetent patients [5]. Even in AIDS patients, it was only isolated on rare occasions. In a retrospective analysis by Butler et al. [6], the rate of isolation of* M. celatum* was determined by examination of 13,530 laboratory isolates over a period of 5 years.* M. celatum* was found in 24 isolates from 17 different patients. The rate of isolation of* M. celatum* types 1 and 2 was only 0.1%, and the rate of isolation of* M. celatum* type 1, which is the type responsible for discrepant reactions with genetic probes, is only 0.05%. A subsequent review including type 3 has not been performed. It is also noted that patients with CVID have not been shown to have increased risk of infection from* Mycobacterium* species [7].

In our case,* M. tuberculosis* was diagnosed based on the direct detection of ribosomal RNA by NAAT. Currently, 2 NAATs, enhanced amplified* Mycobacterium tuberculosis* Direct Test and Amplicor* Mycobacterium tuberculosis* Test (Roche Diagnostics, Basel, Switzerland), have been approved by the US Food and Drug Administration for testing respiratory specimens that are smear-positive for acid-fast bacilli [8]. These commercially available genetic probes are short oligonucleotide sequences and the sequence that is within the target 16S rDNA region for these probes contains a limited, homologous region that differs by only a single nucleotide in both* M. tuberculosis* and* M. celatum* type 1 [9]. It has also been shown that these probes are temperature-dependent and the rate of false-positive identification increases outside the temperature range of 60-61°C.

A meta-analysis from 2006 [3] showed pooled NAAT sensitivities and specificities of 96% and 85%, respectively, on smear-positive samples and 66% and 98%, respectively, for smear-negative samples, making it a strong test to exclude TB on smear positive samples and confirm TB on smear negative samples.

Based on these numbers and given the rarity of* M. celatum*, treatment for* M. tuberculosis* and airborne isolation should still be initiated based on NAAT result. However, correct identification, either by final culture results or by high performance liquid chromatography, remains of importance as* M. celatum* is known to have low in vitro susceptibility to many antituberculous medications [10].

Different regimens with combinations of antimycobacterial agents have been proposed, mainly ethambutol and clarithromycin. A third drug is usually added, such as moxifloxacin in our case or rifabutin [11] and isoniazid [12, 13]. Our species was pan-susceptible to all agents including ciprofloxacin, linezolid, rifampin, and trimethoprim/sulfa as well as to the agents that were ultimately used for treatment in the case above.

Newer probes including the INNO-LiPA Mycobacteria (Innogenetics, Ghent, Belgium) and Geno-Type Mycobacterium (Hain Lifescience, Nehren, Germany) have included probes specific for 16 and 13 different mycobacterium species, respectively, including* M. celatum* [14]. However, they also were found to have issues with misidentification of certain species, namely,* M. smegmatis* identified as* M. fortuitum* and* M. thermoresistibile* identified as* M. celatum*. While any test's sensitivity and specificity depend on the population prevalence, with multiple case reports regarding* M. celatum* infection, the last in 2013 also at Henry Ford Hospital in Detroit [15], we argue that the use of probes specific for* M. celatum* could help prevent misdiagnosis, inappropriate treatment, and the emotional hardship incurred in contact tracing of family members as well as healthcare workers for tuberculosis testing (these writers included).

Fortunately,* M. celatum* is only rarely encountered, and the commercial probes currently available remain highly sensitive and specific. Their continued use as a means of rapidly identifying* M. tuberculosis* complex remains clinically relevant at this time, although it is our hope that more specific probes will become a more cost-effective option in the future to prevent misdiagnosis.
